# Assessment of Professionalism among Postgraduate Medical Students: A Cross-Sectional Study from Patna, Bihar

**DOI:** 10.7759/cureus.64368

**Published:** 2024-07-11

**Authors:** Nidhi Prasad, Sundhareshwaran Chandrasekaran, Binay Kumar, Sanjay Kumar

**Affiliations:** 1 Community Medicine, Indira Gandhi Institute of Medical Sciences, Patna, IND; 2 Pathology, Netaji Subhas Medical College and Hospital, Patna, IND

**Keywords:** competency-based assessment, medical education, lamps, postgraduate medical students, professionalism

## Abstract

Background: Professionalism is a core quality which has to be developed and imbibed by medical students. However, training in professionalism was not a part of the medical curriculum until recently when the Competency-Based Medical Education system was introduced for undergraduate medical students in India, by the National Medical Commission. However, there has been no formal training on professionalism, in the curriculum of postgraduate (PG) medical students in India. Therefore, in this study, we have done a quantitative assessment of the attitude towards professionalism among PG medical students.

Methodology: A cross-sectional study was conducted among 200 PG medical students of an autonomous institute in Patna, Bihar over a period of six months using the Learners’ Attitudes on Medical Professionalism Scale (LAMPS) questionnaire. Data entry was done on Microsft Excel 2015 (Microsoft Corporation, Redmond, Washington, United States) and data analysis was done using IBM SPSS Statistics for Windows, Version 26.0 (Released 2019; IBM Corp., Armonk, New York, United States).

Results: Out of 200 study subjects, 55% (n=110) were PG students from clinical departments and 45% (n=90) were from pre-clinical and para-clinical departments; 25.5% (n=51) of them were from first year, 57% (n=114) of them were from second year, and 17.5% (n=35) were from third year PG training. The overall mean score for the LAMPS scale across all domains was found to be 2.78±0.178 (mean±S.D). Clinical PG students (2.99 ± 0.217) received significantly higher scores than pre/paraclinical PG students (2.68 ± 0.176) (T-test value = 10.9262, p < 0.0001).

Conclusion: This study highlights the disparity in professionalism scores between clinical and pre/para-clinical students, suggesting a tailored approach might be beneficial. In order to improve the attitude towards professionalism, structured courses/workshops should be inculcated into the PG training curriculum. Continuous evaluation of professionalism throughout medical training can lead to better preparedness for future professional roles.

## Introduction

The past two decades have seen a significant emphasis on the concept of professionalism in medical education. In today's complex healthcare landscape, there has been a noticeable transition from a doctor-centric outlook to a more public-centred approach with an emphasis on accountability. This shift has been significantly shaped by the pervasive influence of social media. Professionalism is a complex and multidimensional concept, as highlighted by extensive research [1].

Despite ongoing efforts to provide comprehensive definitions of professionalism, there is no universally agreed-upon definition. In essence, it combines moral dedication and fundamental attributes that manifest through explicit actions [2]. The Accreditation Council for Graduate Medical Education (ACGME) defines professionalism as ‘a commitment to perform professional responsibilities, adhere to ethical principles and be sensitive to the diversity of patients’ population’ [3]. The Royal College of Physicians (2005) has defined medical professionalism as a set of values, behaviours, and relationships that underpins the trust the public has in doctors. Medical professionalism comprises clinical competence, communication skills, and a thorough understanding of the ethical and legal aspects of medicine, all underpinned by accountability, altruism, excellence, and integrity [4].

Under the Competency-Based Medical Education (CBME) system for undergraduate medical students in India, the two major aspects of teaching professionalism include explicit teaching and reflection of competency that align with patient and societal needs as well as modern clinical practice [5-8]. There has been a growing focus on teaching, learning, assessment, and evaluation of professionalism as a part of medical training due to the misconduct of certain doctors at their workplace [9]. To add fuel to the fire, there is an increasing trend of violence against doctors, making the workplace uncertain and unsafe [10,11].

We cannot rely on behaviour acquired by observing the role model alone. Given that professionalism constitutes a fundamental competency in both undergraduate and postgraduate medical education, we have the opportunity to foster the development of professional identity among students, equipping them to effectively navigate the multifaceted challenges they may encounter, i.e., it must be context-specific and take into consideration cultural and societal diversity in different regions, the complex clinical scenario that can lead to ethical dilemmas, and the interplay between social and professional relationships influenced by the etiquettes on social media [12-15].

An abundance of publications on medical professionalism have been published in recent years; however, the number of publications from Asia comprises less than 15% [16]. Therefore, in this study, we have assessed the attitudes of postgraduate medical students towards professionalism as it will provide valuable insights to bridge any existing gaps, enhance skills, and ultimately improve the quality of healthcare services through experiential learning and suggested remedial actions. This study aims to assess the attitude toward professionalism among postgraduate (PG) medical students of an autonomous institute in Patna, Bihar.

## Materials and methods

This was a cross-sectional study conducted among 200 postgraduate medical students at Indira Gandhi Institute of Medical Sciences (IGIMS), Patna, India. The study was conducted from May 2023 to November 2023, over a period of six months. The study was approved by the Institutional Ethical Committee of IGIMS (approval letter no. 876/IEC/IGIMS/2023). After obtaining informed consent, data collection was done through online forms. Participant’s confidentiality and anonymity were thoroughly maintained at all stages.

Sample size

Sample size calculation was done using the formula for Likert scale-based study:

[Z_α/2_^2 ^C^2^ /kD^2^] (1+(k-1)l)

where Z_α/2_ = z score corresponding to the level of significance (at 95% confidence interval (p<0.05), it is equal to 1.96), c= Co-efficient of variation, k= number of items in the questionnaire, d= relative margin of error, l= pairwise correlation coefficient between pairs of items.

In 2021, Razul et al. used the Learners’ Attitudes on Medical Professionalism Scale (LAMPS) questionnaire to assess the attitude towards professionalism among medical students and faculty in their study [10]. Using this study as a reference and under the assumption L=0.3 (most of the pairs). C= SD/mean = 0.45 (from the reference study), k=28, d=5%, the minimum sample size needed was calculated to be 101. However, to attain a reliability coefficient of 0.84 with a width of 0.08, we had taken a sample size of 200. Simple random sampling was used to select the study subjects.

Inclusion and exclusion criteria

Regarding the selection criteria, we included all PG medical students from IGIMS, Patna, while we excluded those from the Department of Community Medicine (owing to potential conflict of interest) and individuals unwilling to participate in the study.

Data collection

Data collection was done using the LAMPS questionnaire, which is a standardized, validated, structured questionnaire (Cronbach’s alpha: 0.79) [17]. LAMPS is a self-assessment tool that captures the perception of the attitude for various domains listed under professionalism. Duty/Accountability, Excellence/ Autonomy, Honor/Integrity, Altruism, and Respect were assessed by 28 items. There were seven items for Duty/Accountability, six items for Excellence/Autonomy, and five items each for Honor/Integrity, Altruism, and Respect. For each item, the participants were asked to provide their responses on a five-point Likert Scale where 1 is "strongly disagree" and 5 is "strongly agree". However, the reverse applies where negative statements were used, where 1 is "strongly agree" and 5 is "strongly disagree". The mean score for each domain as well as the overall mean score was calculated.

Data analysis

Data entry was done using Microsoft Excel 2015 (Microsoft Corporation, Redmond, Washington, United States) and data analysis was done using IBM SPSS Statistics for Windows, Version 26.0 (Released 2019; IBM Corp., Armonk, New York, United States). LAMPS score was reported as mean±SD whereas t-test was used to compare the mean score among different subsets of the subjects.

## Results

General characteristics

Responses of 200 students were received through online forms and were checked for completeness. Of these, 44% (n=88) were female and 56% (n=112) were male. The ages ranged from 23 to 50 years with 11.5% of subjects being ≤25 years of age, 63.5% being between 26-30 years of age, and 25% of the subjects being more than 30 years of age. Among the subjects, 55% (n=110) were PG students from clinical departments and 45% (n=90) were from pre-clinical and para-clinical departments. Regarding distribution across years of residency, 25.5% (n=51) were from the first year, 57% (n=114) were from the second year, and 17.5% (n=35) were from the third year of PG training (Table 1).

**Table 1:** Sociodemographic characteristics of the study subjects (N=200) Chi-square test was used to test the association; p-value <0.05 indicates statistical significance. *Since one of the cells has ‘0’ value, chi-square was not calculated.

Sociodemographic factors		1^st^ year (n=51)	2^nd^ year (n=114)	3^rd^ year (n=35)	Chi-square value	P-value
Age	≤25	9	14	0*	-	-
	26-30	36	78	13		
	>30	6	22	22		
Gender	Male	33	55	24	6.593	0.037
	Female	18	59	11		
Branch category	Clinical	30	65	15	3.581	0.466
	Paraclinical	14	38	14		
	Preclinical	7	11	6		
Parents are doctors	Yes	12	24	7	4.891	0.299
	No	39	90	27		

Mean LAMPS score

The overall mean score for the LAMPS scale across all domains among the 200 subjects was found to be 2.78±0.178 (mean±S.D). The overall mean scores in each domain were as follows: Duty/Accountability 2.63±0.166, Excellence/Autonomy 2.88±0.172, Honor/Integrity 2.54±0.197, Altruism 2.99±0.287, and Respect 2.77±0.124. The mean score in each domain was found to be different among PGs in different years of training (Table 2 and Table 3), as well as among PGs of clinical and pre/para-clinical courses (Table 4).

**Table 2:** Comparison of mean scores across different domains among the first and second year postgraduate students t-test was used to compare the mean values * p-value <0.0001 indicates very high level of statistical significance.

Domains	1^st^ year (n=51), mean±SD	2^nd^ year (n=114), mean±SD	T-test value	P value
Duty/Accountability	2.49±0.194	2.65±0.175	5.2461	<0.0001*
Excellence/Autonomy	2.88±0.164	2.85±0.188	0.9840	0.3266
Honor/Integrity	2.51±0.154	2.70±0.188	6.3269	<0.0001*
Altruism	2.77±0.141	3.02±0.156	8.9145	<0.0001*
Respect	2.86±0.176	2.75±0.153	4.0707	<0.0001*
Overall	2.66 ± 0.180	2.94 ± 0.163	9.8700	<0.0001*

**Table 3:** Comparison of mean scores across different domains of LAMPS among second and third year postgraduate students t-test was used to compare the mean values *p value <0.0001 indicates very high level of statistical significance; **p value <0.001 indicates high level of statistical significance LAMPS: Learners’ Attitudes on Medical Professionalism Scale

Domains	2^nd^ year (n=114), mean±SD	3^rd^ year (n=35), mean±SD	T-test value	P value
Duty/Accountability	2.65±0.175	2.79±0.145	4.2986	<0.0001*
Excellence/Autonomy	2.85±0.188	3.02±0.176	4.7477	<0.0001*
Honor/Integrity	2.70±0.188	2.50±0.091	6.0687	<0.0001*
Altruism	3.02±0.156	3.05±0.176	0.9652	0.3360
Respect	2.75±0.153	2.8±0.098	1.8198	0.0708
Overall	2.94 ± 0.163	2.83 ± 0.174	3.4372	<0.001**

**Table 4:** Comparison of mean scores of different domains between clinical and para/pre-clinical PG students t-test was used to compare the mean values *p<0.0001 indicates very high level of significance PG: postgraduate

Domains	Clinical PG students (n=110), mean±SD	Pre/Paraclinical PG students (n=90), mean±SD	T-test value	P value
Duty/Accountability	2.77±0.134	2.54±0.187	10.1131	<0.0001*
Excellence/Autonomy	3.07±0.241	2.75±0.176	12.3610	<0.0001*
Honor/Integrity	2.84±0.176	2.35±0.171	19.8392	<0.0001*
Altruism	3.39±0.121	2.87±0.124	29.9003	<0.0001*
Respect	2.89±0.134	2.66±0.156	11.2137	<0.0001*
Overall	2.99 ± 0.217	2.68±0.176	10.9262	<0.0001*

## Discussion

Medical professionalism encompasses a spectrum of values, beliefs, behaviours, and attitudes that society deems essential for physicians. The LAMPS questionnaire features 28 items categorized into five essential domains: Duty/Accountability, Excellence/Autonomy, Honor/Integrity, Altruism, and Respect. Each domain targets a distinct facet of medical professionalism. For instance, Duty/Accountability addresses actions like acknowledging errors and ensuring proper patient handovers, providing insights into positive or negative professional attitudes. Excellence/Autonomy examines ongoing education and collaborative efforts to enhance patient care. Honor/Integrity emphasizes ethical behaviour, focusing on honesty in patient relations and adherence to research standards. Altruism assesses the prioritization of patient needs over personal interests, reflecting a commitment to patient welfare. Lastly, Respect evaluates the recognition of various roles within the healthcare team and the consideration of patient backgrounds. Together, these domains offer a thorough assessment of professionalism among PG medical students, capturing their responses to diverse professional scenarios.

Medical professionalism lacks a universally standardized definition due to variations in culture and context [18]. However, certain domains or traits have been integrated into its definition. The American Board of Internal Medicine (ABIM) has outlined altruism, accountability, duty, excellence, integrity, honour, and respect as six key components of medical professionalism [19]. These components formed the basis for Al-Eraki's development and validation of the LAMPS [17]. The LAMPS assesses not just students' attitudes but also evaluates scores across its domains and items, which can pinpoint learning deficiencies in specific aspects of professionalism. Recognizing these gaps and misunderstandings in professionalism among students and assisting them in addressing these areas is crucial for nurturing professionalism [20] and meeting the societal responsibilities of a medical school [21].

The findings from this study shed light on several aspects of professionalism among PG medical students. Furthermore, the mean scores across different domains of professionalism provide valuable insights into the varied aspects of professionalism among PG medical students. In terms of PG training year-wise comparisons, the study found that second-year PG students consistently exhibited higher mean scores across various domains compared to their first-year counterparts. This trend suggests a progressive development of professional attitudes and behaviours as students advance through their training, with a notable emphasis on duty, honour, altruism, and respect. However, comparisons between second and third-year PG students revealed contrasting findings. While third-year students demonstrated higher scores in duty/accountability and excellence/autonomy domains, they scored lower in Honor/Integrity and respect domains compared to their second-year counterparts. This could be attributed to several factors such as, but not limited to, burnout, facing challenging and complex clinical scenarios in their final year of training and an increased academic responsibility due to thesis submission and exam preparation.

Additionally, the comparison between clinical and pre/paraclinical PG students highlighted significant differences. Clinical PG students consistently scored higher across all domains, indicating a stronger sense of duty, excellence, honour, altruism, and respect compared to their pre/paraclinical counterparts. This could be due to several factors such as direct patient interaction, professional role-modelling, patient-centred academic learning and skills development exhibited by clinical PG students.

Rasul et al.’s study inferred significant differences among attitudes of the first and final year medical students in the domains of Excellence/Autonomy and Altruism, like our study [10]. Honor/Integrity was the most valued trait and Excellence/Autonomy was the lowest-scored domain of medical professionalism. However, in our study, Excellence/Autonomy was the highest-scored domain and Honor/Integrity was the lowest-scored domain.

Desai et al, in their review article, highlight that educating medical students on professionalism and ethics can enhance their understanding of obligations and ethical duties [22]. The CBME training for undergraduate students, which includes foundational courses, is designed to provide essential skills and knowledge, forming the core of their medical education. Key elements include orientation to the medical profession, communication and IT skills, learning techniques, community health, professional ethics, and first aid training. This holistic approach ensures that students are well-prepared for both the academic and professional challenges of their medical careers. The AETCOM (Attitude, Ethics, and Communication) module is a component of the CBME curriculum and it aims at cultivating ethical values and effective communication skills among future physicians, thereby preparing them to practice medicine with a strong emphasis on professionalism. Amarantha et al. found significant differences in mean attitude scores between students exposed to formal AETCOM training and those who were not [23]. This indicates that formal training in AETCOM competencies has a positive effect in shaping the professional attitudes of undergraduate medical students. However, the PG teaching curriculum currently lacks specific areas of focus on professionalism.

Professionalism and medical ethics go hand in hand. To attain a state of professional excellence in the field of medicine, one should have a crisp knowledge of medical ethics as well. Lee et al., in their study, found that there was inadequate knowledge about professionalism as well as medical ethics among medical students and residents, in Korea [24]. Similar results were also inferred by Rabbani et al. (Pakistan), Rafique et al. (Saudi Arabia), Blue et al. (United States), Spiwak et al. (Canada), Jahan et al. (Oman), and McGurgan et al. (Australia) [25-30]. These findings align with the findings of our study, indicating a universal lack of a structured curriculum to teach and evaluate professionalism and medical ethics among medical students and medical residents.

Overall, our study underscores the dynamic nature of attitudes towards different domains of professionalism among PG medical students and emphasizes the importance of interventions to foster and reinforce positive professional attitudes and behaviours throughout their training. The strength of this study lies in its comprehensive assessment of professionalism attitudes among PG medical students, providing valuable insights into the development of these attitudes throughout the course of PG medical education in India. Additionally, this is one of the first quantitative assessments of professionalism across five major domains among PG students from the Indian subcontinent, contributing significantly to the literature in this area. However, the study's findings have limited generalizability due to it being conducted at a single institution, which may not reflect the demographics and training environments of other medical institutions in the country. Additionally, the reliance on self-reported data introduces the possibility of bias inherent in questionnaire-based studies.

## Conclusions

In the initial stages of residency, PG students transition from a predominantly academic environment to the practical, hands-on demands of clinical practice. This phase is vital for cultivating foundational professional attitudes and behaviours. As residents advance in their training, they encounter greater responsibilities and more complex clinical scenarios, which continue to influence and refine their professional attitudes. This study offers valuable insights into the attitudes toward various domains of professionalism among PG medical students, highlighting the variations across different years of residency training as well as different fields of residency training. The study revealed that scores increased with increasing years of residency training in the majority of domains, with second-year PG students consistently exhibiting higher mean scores compared to their first-year counterparts, and third-year PG students exhibiting higher mean scores compared to their second-year counterparts, suggesting a progressive development of professional attitudes as students advance in their training. Additionally, clinical PG students consistently scored higher across all domains compared to their pre/para-clinical counterparts, indicating a stronger emphasis on professionalism among those directly involved in patient care.

These findings emphasize the need for structured courses or workshops to enhance professionalism among medical residents. Integrating comprehensive professionalism training into the curriculum is crucial for developing physicians capable of upholding the highest professional standards. A tailored course with assessments, similar to the AETCOM module in the CBME curriculum for undergraduates, should be mandated for PG medical students, focusing on key domains of professionalism. Future research should include qualitative studies to identify specific gaps in professionalism attitudes, providing a deeper understanding of underlying issues. Furthermore, longitudinal studies are recommended to track the development of professional attitudes and behaviours over time, offering insights into their progression through residency training.

## References

[1] Hilton SR, Slotnick HB (2005). Proto-professionalism: how professionalisation occurs across the continuum of medical education. Med Educ.

[2] Birden H, Glass N, Wilson I, Harrison M, Usherwood T, Nass D (2014). Defining professionalism in medical education: a systematic review. Med Teach.

[3] Kelly AM, Mullan PB, Gruppen LD (2016). The evolution of professionalism in medicine and radiology. Acad Radiol.

[4] Seif-Farshad M, Bazmi S, Amiri F, Fattahi F, Kiani M (2016). Knowledge of medical professionalism in medical students and physicians at Shahid Beheshti University of Medical Sciences and affiliated hospitals-Iran. Medicine (Baltimore).

[5] (2018). AETCOM Handbook.

[6] Shrivastava SR, Shrivastava PS (2018). How to successfully implement competency-based medical education in India. Educ Health Prof.

[7] Goddard AF, Patel M (2021). The changing face of medical professionalism and the impact of COVID-19. Lancet.

[8] Horlick M, Masterton D, Kalet A (2006). Learning skills of professionalism: a student led professionalism curriculum. Med Educ Online.

[9] Kaur A, Ahamed F, Sengupta P, Majhi J, Ghosh T (2020). Pattern of workplace violence against doctors practising modern medicine and the subsequent impact on patient care, in India. PLoS One.

[10] Rasul S, Zahid Bashir M, Saleem S, Tahir S, Rasheed A, Ali Sabir M (2021). Assessment of medical professionalism among students and faculty members of Shalamar Medical and Dental College, Lahore. J Adv Med Educ Prof.

[11] Papadakis MA, Hodgson CS, Teherani A, Kohatsu ND (2004). Unprofessional behavior in medical school is associated with subsequent disciplinary action by a state medical board. Acad Med.

[12] Morihara SK, Jackson DS, Chun MB (2013). Making the professionalism curriculum for undergraduate medical education more relevant. Med Teach.

[13] Jameel A, Noor SM, Ayub S, Tekian A (2013). Why is teaching professionalism essential in residency programmes?. Med Educ.

[14] Chandratilake M, McAleer S, Gibson J (2012). Cultural similarities and differences in medical professionalism: a multi-region study. Med Educ.

[15] O'Sullivan H, van Mook W, Fewtrell R, Wass V (2012). Integrating professionalism into the curriculum: AMEE Guide No. 61. Med Teach.

[16] Song X, Jiang N, Li H, Ding N, Wen D (2021). Medical professionalism research characteristics and hotspots: a 10-year bibliometric analysis of publications from 2010 to 2019. Scientometrics.

[17] Al-Eraky MM, Chandratilake M, Wajid G, Donkers J, van Merrienboer J (2013). Medical professionalism: development and validation of the Arabian LAMPS. Med Teach.

[18] Dubbai H, Adelstein BA, Taylor S, Shulruf B (2019). Definition of professionalism and tools for assessing professionalism in pharmacy practice: a systemic review. J Educ Eval Health Prof.

[19] Kimball HR (1995). Credentials misrepresentation: another challenge to professionalism. Ann Intern Med.

[20] Hur Y (2009). Are there gaps between medical students and professors in the perception of students' professionalism level?--Secondary publication. Yonsei Med J.

[21] Woollard B, Boelen C (2012). Seeking impact of medical schools on health: meeting the challenges of social accountability. Med Educ.

[22] Desai MK, Kapadia JD (2022). Medical professionalism and ethics. J Pharmacol Pharmacother.

[23] Amarantha DR, Noor T, Swanand SP (2022). Effect of education in AETCOM competencies in shaping the professional attitudes of medical students. South East Asian J Med Educ.

[24] Lee JK, Lee SY, Hahm SK (1995). Understanding of medical ethics among medical students and residents. Korean J Med Educ.

[25] Rabbani MW, Mujtaba M, Naeem F, Ullah E (2014). Knowledge and awareness of medical ethics among medical graduates from two medical colleges in Lahore, Pakistan. Rawal Med J.

[26] Rafique M, Nuzhat A, Enani MA (2017). Professionalism in medical education perspectives of medical students and faculty. MedEdPublish.

[27] Blue AV, Crandall S, Nowacek G, Luecht R, Chauvin S, Swick H (2009). Assessment of matriculating medical students' knowledge and attitudes towards professionalism. Med Teach.

[28] Spiwak R, Mullins M, Isaak C, Barakat S, Chateau D, Sareen J (2014). Medical students' and postgraduate residents' observations of professionalism. Educ Health (Abingdon).

[29] Jahan F, Siddiqui MA, Al Zadjali NM, Qasim R (2016). Recognition of core elements of medical professionalism among medical students and faculty members. Oman Med J.

[30] McGurgan P, Calvert KL, Narula K, Celenza A, Nathan EA, Jorm C (2020). Medical students' opinions on professional behaviours: the professionalism of medical students' (PoMS) study. Med Teach.

